# Supplementary figures and images for: Evidence for RNA or protein transport from somatic tissues to the male reproductive tract in mouse
Source: eLife. 2023 Mar 27;12:e77733. doi: 10.7554/eLife.77733 (PMC10079288; doi:10.7554/eLife.77733)

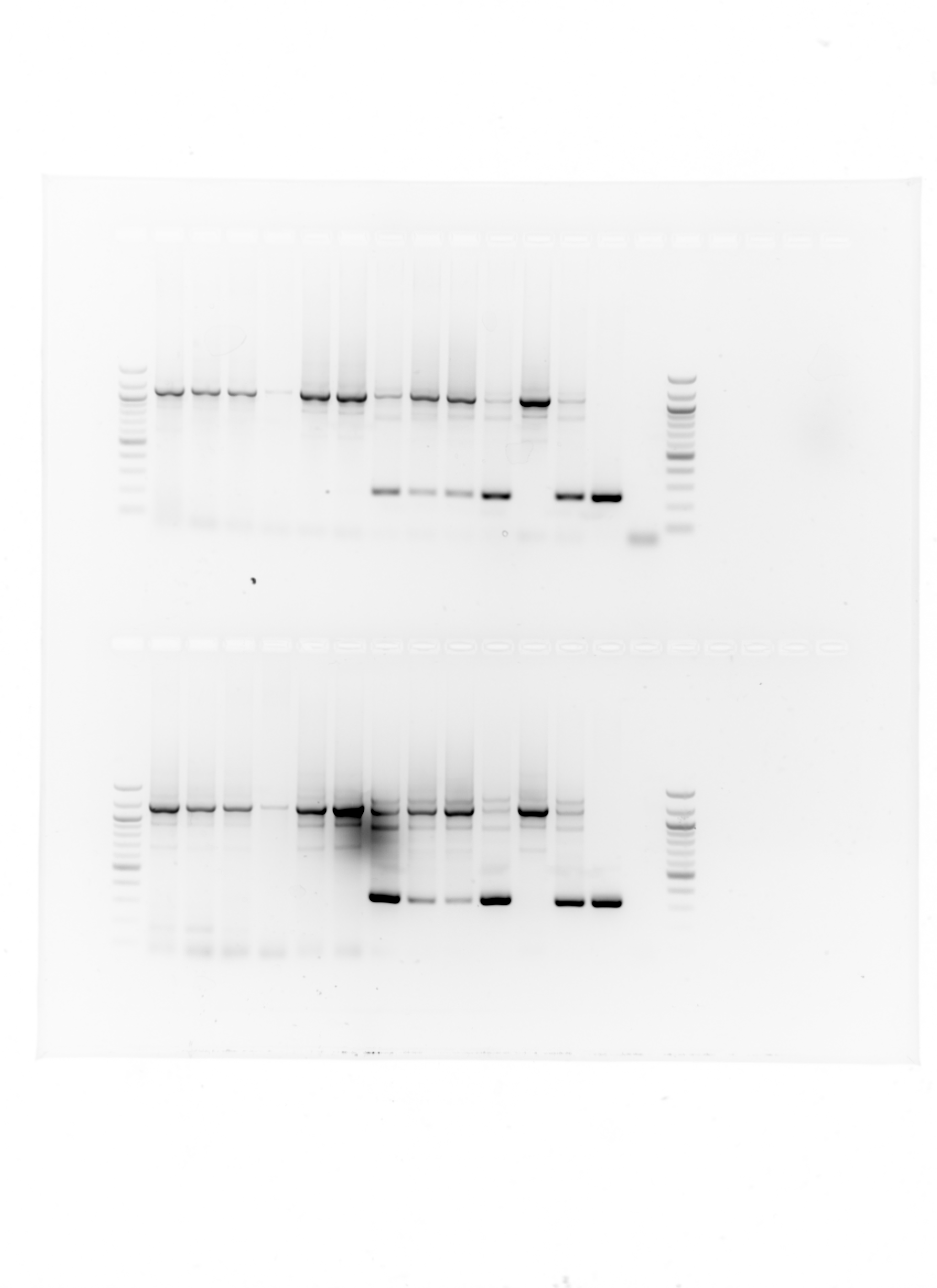

Supplement: Source data 1. [file elife-77733-data1.zip › Rinaldi_Fig3E_source.tif]

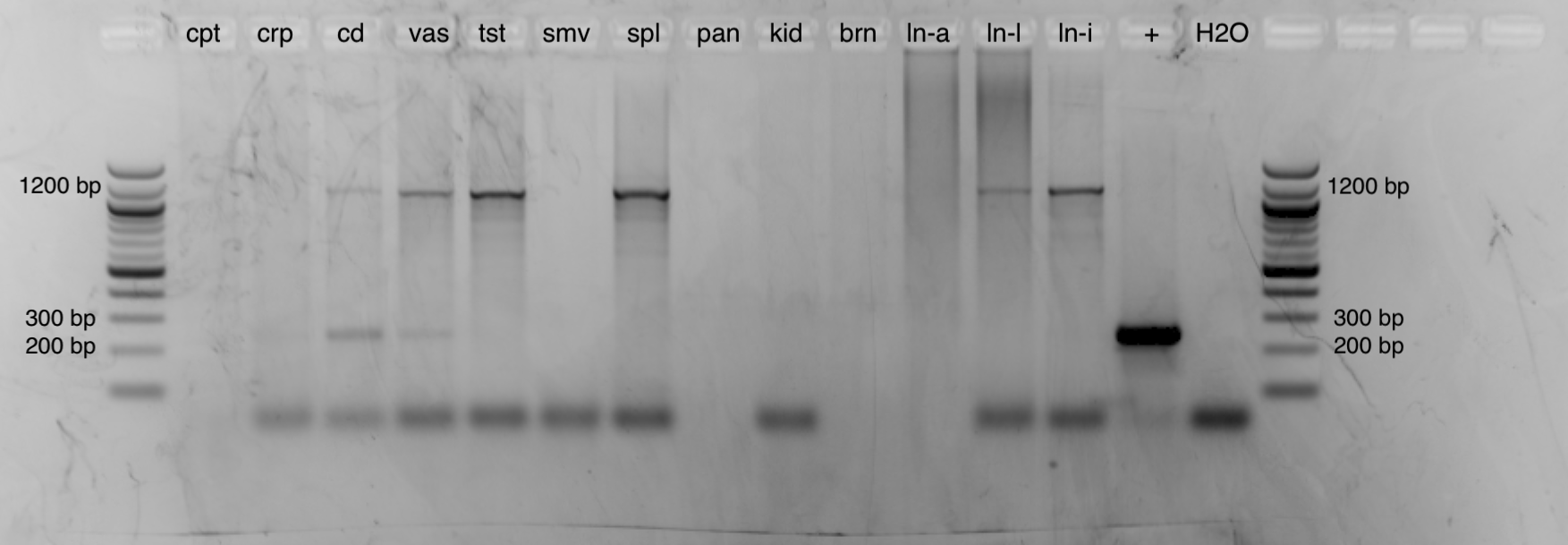

Supplement: Source data 1. [file elife-77733-data1.zip › Rinaldi_Fig4S1C_source.tif]

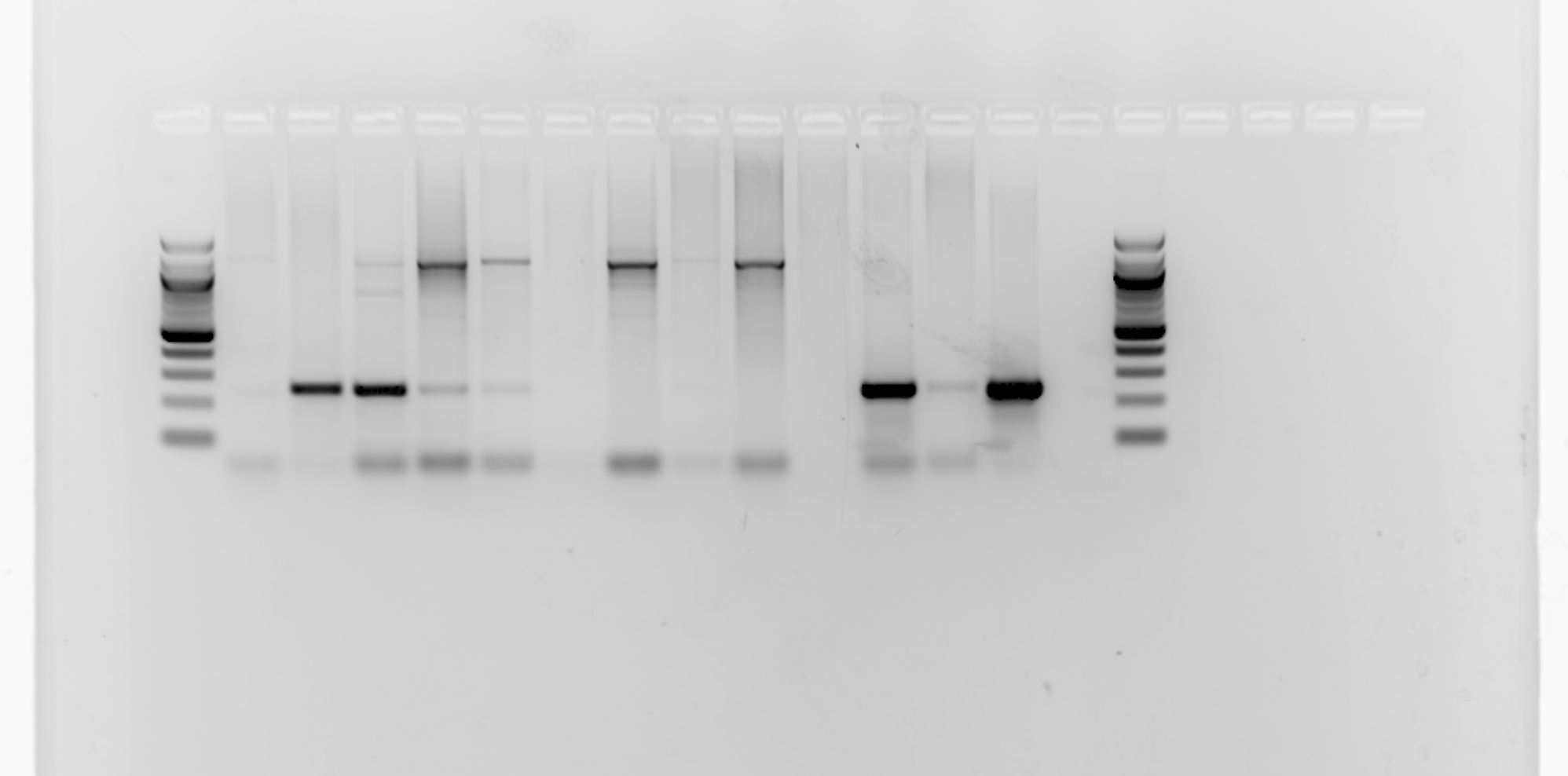

Supplement: Source data 1. [file elife-77733-data1.zip › Rinaldi_Fig5C_source.tif]

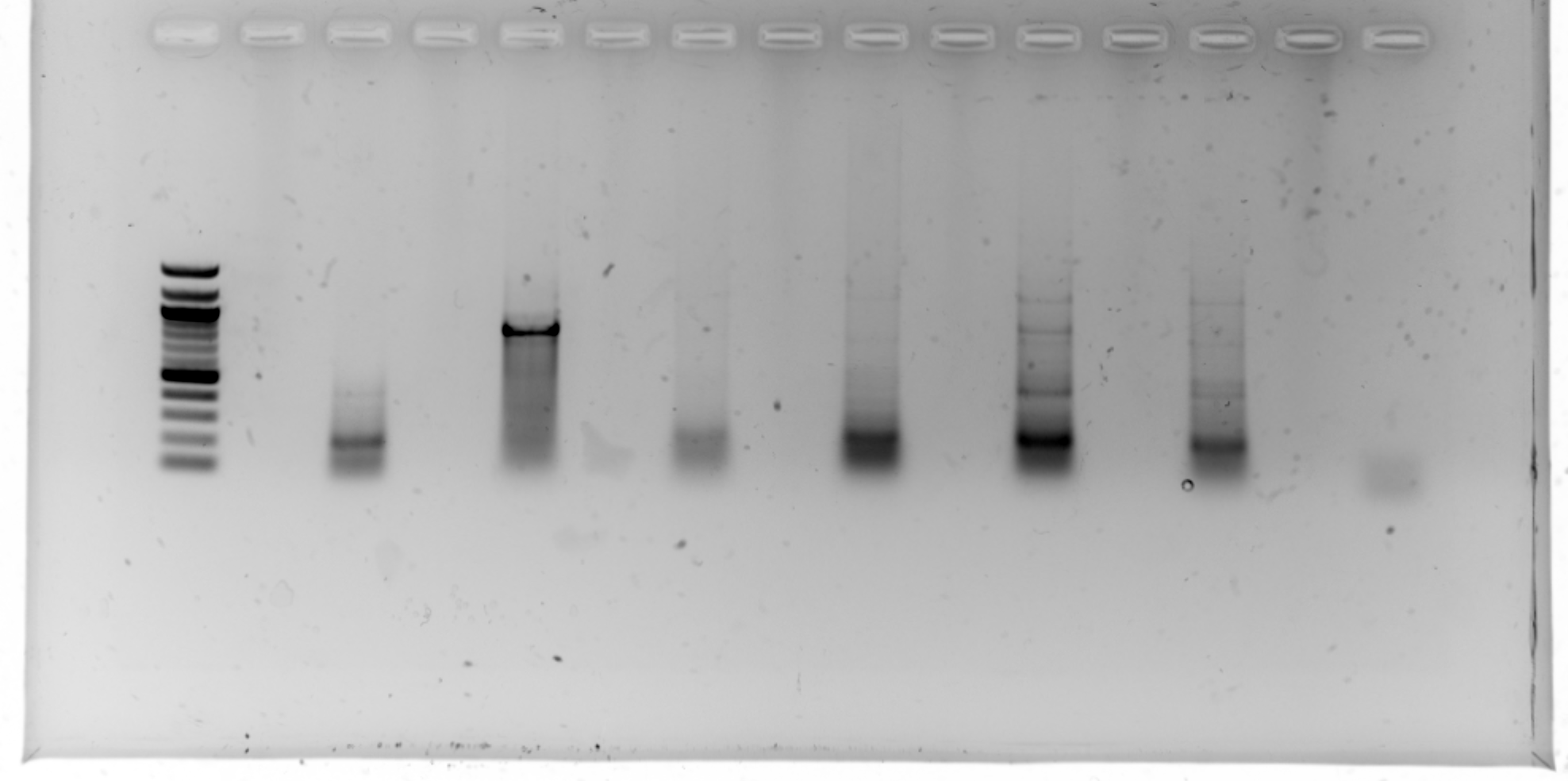

Supplement: Source data 1. [file elife-77733-data1.zip › Rinaldi_Fig5S1C_source.tif]

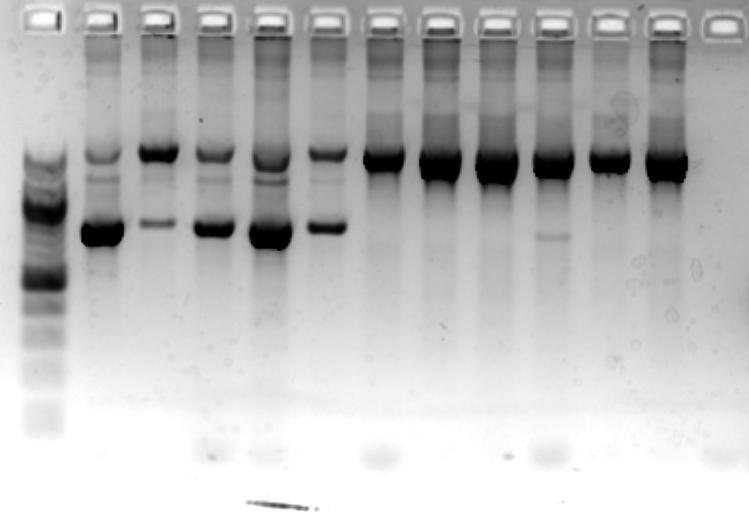

Supplement: Source data 1. [file elife-77733-data1.zip › Rinaldi_Fig6C_source.tif]

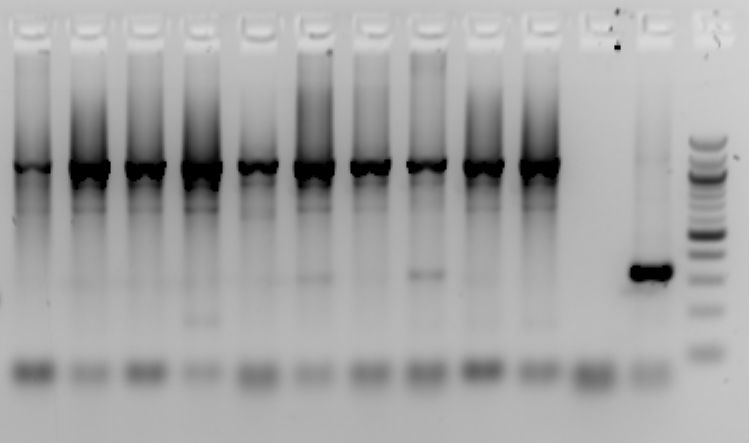

Supplement: Source data 1. [file elife-77733-data1.zip › Rinaldi_Fig6F_source.tif]

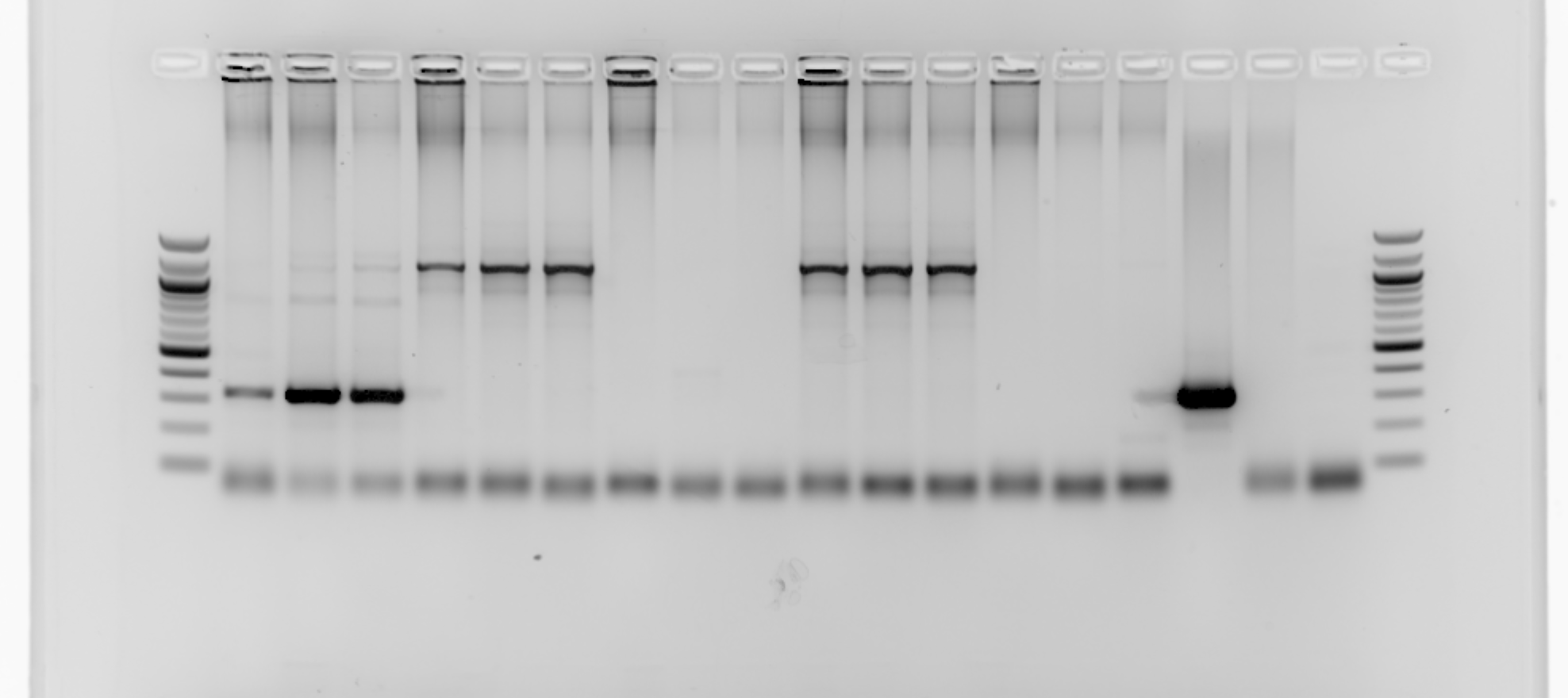

Supplement: Source data 1. [file elife-77733-data1.zip › Rinaldi_Fig6S1C_source.tif]

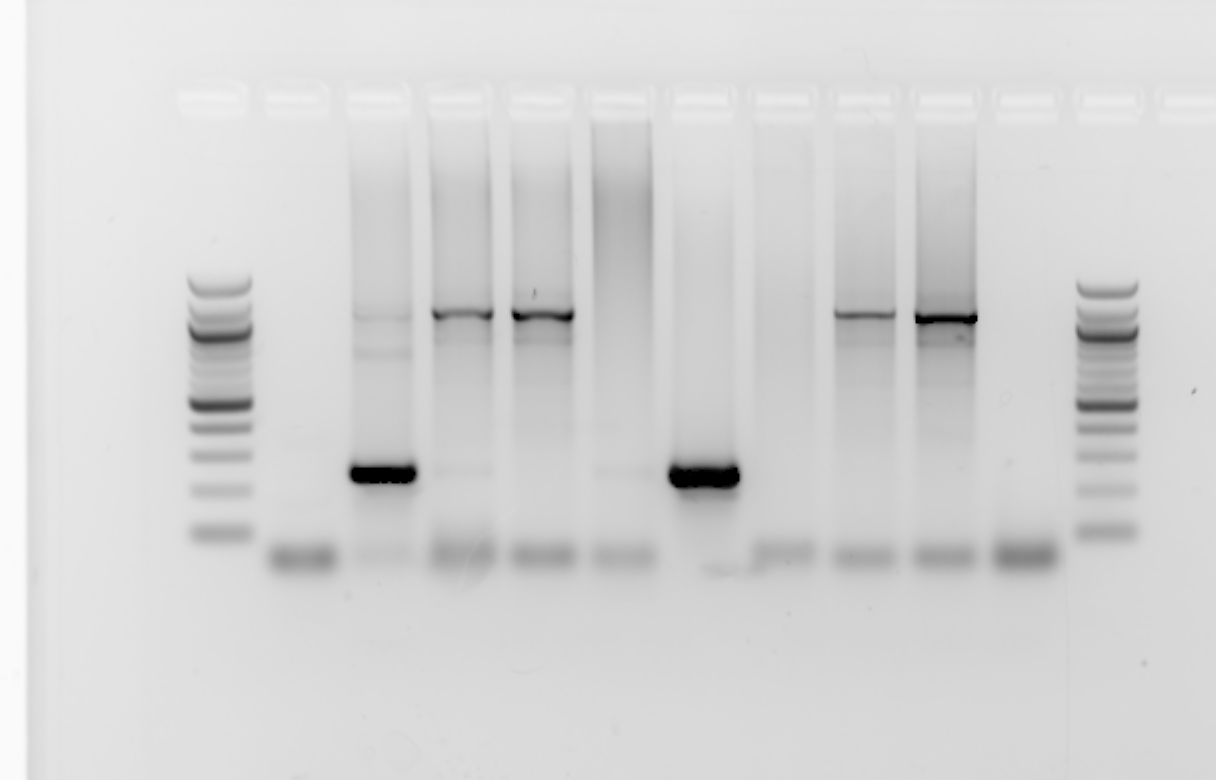

Supplement: Source data 1. [file elife-77733-data1.zip › Rinaldi_Fig6S1F_source.tif]

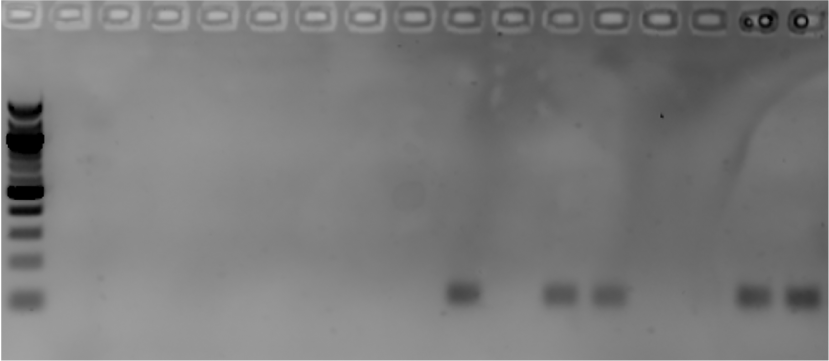

Supplement: Source data 1. [file elife-77733-data1.zip › Rinaldi_Fig6S2_source.tif]
